# Quantification of Alzheimer disease neuropathology using tissue microarrays

**DOI:** 10.1093/jnen/nlaf064

**Published:** 2025-06-24

**Authors:** Hoang-Tuong Nguyen-Hao, Jie Liu, Mario Novelli, Dhiraj Maskey, Raymond S Schwartz, Oscar Harari, Greg T Sutherland

**Affiliations:** Charles Perkins Centre and School of Medical Sciences, Faculty of Medicine and Health, The University of Sydney, Camperdown, NSW, Australia; Charles Perkins Centre and School of Medical Sciences, Faculty of Medicine and Health, The University of Sydney, Camperdown, NSW, Australia; Charles Perkins Centre and School of Medical Sciences, Faculty of Medicine and Health, The University of Sydney, Camperdown, NSW, Australia; Charles Perkins Centre and School of Medical Sciences, Faculty of Medicine and Health, The University of Sydney, Camperdown, NSW, Australia; Charles Perkins Centre and School of Medical Sciences, Faculty of Medicine and Health, The University of Sydney, Camperdown, NSW, Australia; Department of Neurology, Neurological Research Institute, Ohio State University, Columbus, OH, United States; Charles Perkins Centre and School of Medical Sciences, Faculty of Medicine and Health, The University of Sydney, Camperdown, NSW, Australia

**Keywords:** Alzheimer’s disease, hallmark pathologies, multiplex immunofluorescence, tissue microarray

## Abstract

Alzheimer’s disease (AD) is characterized by the hallmark pathologies of β-amyloid plaques and neurofibrillary tangles (NFTs), which vary across brain regions and between affected individuals. As a rapid research method to quantify hallmark pathologies and their relationships with the surrounding cytoarchitecture across the brain, we trialed tissue microarrays (TMAs) in combination with multiplex immunofluorescence and semi-automated image analysis. We assayed the hallmark pathologies across grey and white matter sites of 11 different brain regions and quantified astrocytes, microglia, and neurons in four AD cases and four age- and gender-matched controls. The results demonstrated that 2 mm cores differentiated AD-affected individuals from healthy donors. Regional amyloid loads corroborated the established spread from the neocortex to allocortex, pontine, and occasionally cerebellar regions. Similarly, NFT counts matched the pattern predicted by Braak staging. Contrasting with the two hallmark pathologies, regional cell numbers were similar between cases and controls although cell counts were affected by variable staining quality. These results suggest that TMAs in combination with multiplex immunofluorescence and image analysis offer a powerful research tool for the rapid assessment of brain-wide AD neuropathological change in postmortem tissue. This quick assessment allows an informed selection of regions for more comprehensive investigations.

## INTRODUCTION

Alzheimer’s disease (AD) is a neurodegenerative disease characterized by progressive loss of memory and cognitive abilities. Historically, the disease has been diagnosed by the combined qualification of two hallmark pathologies: amyloid-beta (Aβ) plaques within the brain parenchyma and intra-neuronal neurofibrillary tangles (NFTs).^1^ The spread of these pathologies throughout the brain has been histopathologically characterized with Aβ accumulation following an overarching inward trajectory from ventral cortical surfaces to the remaining structures, including the thalamus, brainstem, and cerebellum.^2^^,^^3^ In contrast, tau pathology spreads from allocortical structures to the neocortex, and more closely resembles symptomology such as memory deficits, cognitive decline, and motor impairments.^2^^,^^4^

The employment of molecular techniques has led to a greater appreciation of glial cells and their interactions with what had been a largely neuron-centric model of AD pathogenesis. Bulk tissue, single nucleus RNA sequencing, genome-wide association studies, and their meta-analyses have established glial cells as critical players in the progression of hallmark pathologies.^5–7^ Astrocytes are now known to be a functionally, morphologically, and spatially diverse group of cells that mediate a wide range of functions for brain homeostasis, including synaptic maintenance through recycling of neurotransmitters, neuronal trophic supports, neurovascular coupling, and regulation of both influx and efflux across the blood-brain barrier (BBB).^8^^,^^9^

Similarly, diverse subtypes of microglia, the resident cells of the central nervous system (CNS) are being identified.^8^^,^^10^^,^^11^ Under Aβ insults, these cells respond by upregulating Aβ-clearance molecules such as apolipoprotein-E (APOE), triggering receptors expressed on myeloid cells 2 (TREM2), neprilysin (NEP), and insulin-degrading enzyme (IDE), to ameliorate the pathological loads. However, the late AD disease stages see these neuroprotective effects wane in favor of more neurotoxic phenotypes that promote the production rather than clearance of these hallmarks.^12–14^

The long disease progression of AD where symptoms appear decades after disease hallmarks start to form^15^ makes the study of pathomechanisms challenging. The timing of events leading to pathological deposits and cell death remains fragmented as histopathological human studies are performed using postmortem tissues, resulting in temporally restricted snapshots of the terminal disease stage. We have previously suggested that exploration of the brain regions last affected by Alzheimer’s disease neuropathological change (ADNC) may harbor clues to the earliest pathomechanisms and particularly those associated with reversible neuron changes.^5^^,^^16^ In these regions, neuropathological quantifications would complement molecular data and enable interpretations of processes that are plaque- or NFT-dependent, or potentially independent of pathology altogether. A recent analysis of the moderately-affected middle frontal gyrus in a large cohort of pathologically confirmed AD cases and controls suggests that this is indeed the case.^17^ An extension of this idea is that comparing molecular signatures across several incrementally more affected regions within each AD brain allows the natural course of the disease to be modelled directly in the human brain. However, as ADNC is known to vary among individuals,^18^ these incrementally affected brain regions need to be empirically determined.

Stereological approaches are the gold standard for quantifying cells and pathology in the brain. Given the size of the human brain and resource-intensive nature of these approaches, global quantitative neuropathology studies in AD have been rare^19^^,^^20^ or limited to very few regions.^5^ This means that the semi-quantitative schemas predicting the spread of ADNC have rarely been tested for their generalizability. To address this issue, we have employed tissue microarrays (TMAs) as a rapid platform for the global quantitative assessment of AD pathologies across the human brain. TMAs have been used extensively in cancer research to assay multiple donors simultaneously, and this same approach has been employed in postmortem brain tissue^21^ and has subsequently been employed in the study of Huntington disease^22^ and AD.^23^ In 2006, a group saw the value of alternatively employing TMAs to cover multiple brain regions in the same individual for the study of neurodegenerative diseases.^24^^,^^25^ This group performed singleplex immunohistochemical (IHC) stains of multiple, adjacent tissue sections with semi-quantitation of pathology following manual assessment under light microscopy, and limited sampling consistent with their labour-intensive approach. Our current work builds on these efforts but uses multiplex immunofluorescence (IF) to capture all the cell types and hallmark pathologies on a single section, thereby maintaining accurate spatial relationships. Furthermore, quantifiable data were obtained using machine learning algorithms on digitalized images, removing or minimizing single rater variability between manual sampling sessions. Importantly, amplification inherent to IF increases the signal-to-noise ratio relative to IHC. This proof-of-concept study shows that TMAs are an effective research method to gain a rapid but accurate overview of ADNC within and between individuals. This can then be used to inform region selection for more detailed studies towards a better understanding of pathomechanisms and new therapeutic targets.

## METHODS

### Tissue acquisition

Formalin-fixed paraffin-embedded tissues from eight age- and gender-matched individuals (4 AD: 4 controls) were provided by the NSW Brain Tissue Resource Centre (BTRC) with BTRC Scientific Advisory Committee and the University of Sydney Human Ethics Committee (2018/HE000477) approval. ‘Archival tissue’ denotes tissue held in 10% formalin for a period exceeding 10 years before embedding for this study (Table 1). Pathological assessments were carried out by an experienced BTRC neuropathologist according to the National Institute on Aging-Alzheimer’s Association, with all four diseased donors having a high probability of AD (A3B3C3).^26^ Controls had Aβ or tau pathologies corresponding to A1 and B1, respectively, but exhibited no lifetime cognitive deficits. BTRC takes 16 standard blocks from each fixed tissue hemisphere for general diagnostic purposes and as the commonly requested regions for research.^27^ 11/16 blocks were chosen here to represent areas likely to be incrementally affected by ADNC according to the semi-quantitative schema,^2^^,^^3^ ie, immunolabelled plaques. Given the paucity of postmortem human tissue and inevitable secondary damage to older and more brittle donor blocks from the coring mechanism, cores were taken from the periphery to minimize potential damage to the bulk of tissue blocks (Figure S1). TMAs were constructed with 35× 2 mm cores made up of seven neocortical regions: prefrontal cortex ([PFC]; 4 cores; 2 grey matter [GM] cores [Figure S2A], an adjacent white matter [WM] core and a deep WM core [for two regions only]), parietal cortex ([PC]; 4 cores), primary motor cortex ([PMC]; 4 cores), occipital cortex ([OC]; 3 cores), superior ([STG]; 3 cores) and inferior ([ITG]; 3 cores) temporal gyri and anterior cingulate cortex ([ACC]; 3 cores); the (allocortical) entorhinal cortex ([EC]; 2 cores; [Figure S2B]); pons (sampled at the corticospinal tract, posterior to corticospinal tract, and superficial to the fourth ventricle; 3 cores in total) (Figure S2C) and the cerebellar vermis ([CV]; 3 cores) and cerebellar cortex ([C]; 3 cores; [Figure S2D]). The entorhinal region was particularly chosen to eliminate such effects to the brain bank highly requested hippocampal proper.

**Table 1.** Cohort data summary.

ID	Sex	Status	Age of death	Brain pH	**PMI** ^a^ **(hrs)**	**ABC score** ^b^	APOE genotype
AD1	Female	AD	78	6.60	6	A3B3C3	E4/E4
AD2	Male	AD	74	6.55	19	A3B3C3	E3/E4
AD3	Male	AD	80	5.84	8	A3B3C3	E3/E4
AD4	Female	AD	90	6.33	19	A3B3C3	E3/E3
C1	Male	Control	73	6.52	9	A1B1C0	E3/E3
C2	Male	Control	80	6.50	12	A0B0C0	E3/E3
C3	Female	Control	91	6.15	12	A0B1C0	E3/E3
C4	Female	Control	77	6.17	< 24	A0B1C0	E3/E4

a PMI: postmortem interval.

b ABC score^21^: neuropathological assessment of AD based on severity of AD pathology—a combination of:

“A” measures Aβ according to Thal et al. (2002).^26^ 0 = no pathology, 1 = phases 1 + 2, 2 = phase 3, 3 = phases 4 + 5.

“B” measures NFTs according to Braak and Braak (1991).^2^ 0 = no pathology, 1 = stages I + II, 2 = stages III + IV, 3 = stages V + VI.

“C” measures neuritic plaques according to CERAD (Consortium to Establish a Registry for Alzheimer’s Disease) as defined by Mirra et al.^1^ 0 = no pathology, 1 = sparse, 2 = moderate, 3 = frequent neuritic plaques.

### TMA construction

TMAs were constructed with the recipient-block method with 2 mm diameter tissue plugs using a TMA Master II (3DHisTech Ltd; courtesy of the New South Wales Statewide Biobank). For neocortical blocks, donor tissues were double cored in a staggered manner across the cortical grey matter of the gyrus to capture all six neocortical layers (Figure S2B). TMA blocks were subsequently annealed using four cycles of 1-hour heating at 37 °C and 1-hour 4 °C refrigeration.^28^ Finally, blocks were incubated at 46 °C for 1 hour followed by rehydration in a water bath prior to sectioning into 7-µm slide for multiplex IF (mIF) (HM 325 Manual Microtome, ThermoScientific).

### Multiplex immunofluorescence

TMA slides were initially dewaxed and rehydrated via serial solutions of alcohol. Slides were additionally incubated with 10% neutral buffered formalin for 10 minutes to improve tissue adhesion, followed by 15-minute incubation with 90% formic acid to increase Aβ antigenicity. Non-specific heat-induced epitope retrieval (HIER) was performed by pressure cooking (Decloaking Chamber NxGen, Biocare Medical) in AR6 Buffer (Akoya Biosciences, Marlborough, MA, USA) at 110 °C and 5 psi for 10 minutes.

Manual mIF was performed according to the tyramide substrate amplification protocol from Akoya Bioscience. In brief, target proteins were labelled by sequential incubations with antigen-specific primary antibodies, anti-mouse/rabbit secondary antibodies, and finally, Opal tyramide fluorophores. HIER was performed for 10 minutes after each labelling to elute the bound antibodies, but not the fluorophore. Astrocytes, microglia, tau, and Aβ were sequentially labelled using anti-GFAP (rabbit, Agilent Z033401. 1:2000), anti-Iba1 (rabbit, Wako 019-19741, 1:500), anti-total tau (t-tau) (mouse, Agilent A0024-01, 1:2000), and anti-β-Amyloid 1-16 (mouse, BioLegend 803004, 1:500). Anti-T-tau staining over the commonly used phospho-tau antibody staining (p-tau; typically, AT8) was performed because of the greater immunoreactivity of the former antibody to label neuropil threads (NTs), neuritic plaques (NPs), NFTs and other neurofibrillary pathology (Figure S3). Primary antibodies were subsequently labelled with Opal 690, 520, 570, and 620, respectively. Following the final HIER stripping, slides were incubated with 4’,6-diamidino-2-phenylindole (DAPI) and mounted with ProLong Diamond Antifade mounting agent (Invitrogen, Thermo Fischer Scientific).

### Immunohistochemistry

NeuN visualization was carried out with 3,3’-diaminobenzidine (DAB) using an adjacent serial section to mIF. After rehydration and HIER, slides were treated for endogenous peroxidase followed with non-specific protein blocking using 3% hydrogen peroxide in methanol and 10% normal horse serum (LOT: 2054010, Gibco) for 10 minutes each. Slides were subsequently incubated with anti-NeuN antibody (Merck ABN78, 1:1000) overnight at 4 °C followed by an HRP-conjugated secondary antibody (DAKO) for 10 minutes.

### Image capture and autofluorescence control

Image acquisitions for both mIF and chromogenic slides were carried out using the PhenoImager Fusion (Akoya Biosciences; courtesy of the Melanoma Institute of Australia) at 20× magnification and a resolution of 0.50 µm/pixel. Focal points were automated, and exposures for each channel were individually assessed for each TMA to account for variability in staining quality. Post-scanning, images were further controlled for autofluorescence and signal crosstalk with inForm (Akoya Bioscience) using a custom Opal monoplex and brain-specific autofluorescence library.

### Image analyses

**Figure 1.**
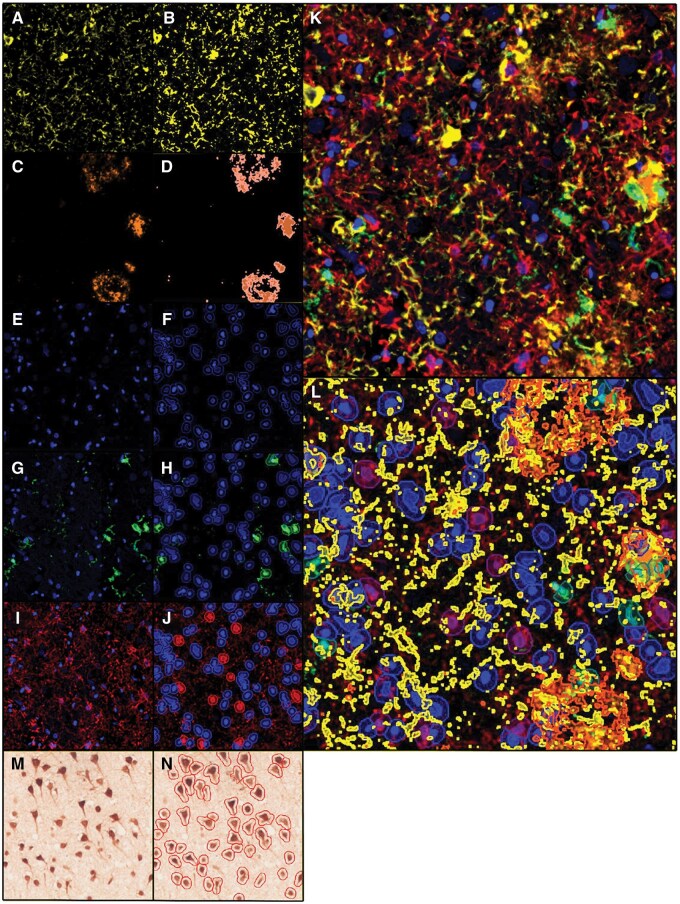
Image analysis workflow on QuPath of a PC core tissue with Alzheimer disease neuropathological changes. (A)-(H): Single-channel view and pixel quantification of AD pathologies: (A) and (B) T-tau and (C) and (D) Aβ 1-16 using full pixel resolution (0.5 µm/pixel). (E) and (F) Nuclei labelled by the DNA marker, DAPI and (G) and (H) their segmentation, and single-channel views and classification and of segmented d.i-ii) Iba1^+^ microglia, (I) and (J) GFAP^+^ astrocytes. (K) Merged mIF and the (L) resultant detections after sequential pixel and object classifications. (M) DAB chromogenic staining of NeuN^+^ neurons on a serial section to and (N) their segmentation using optical density sum.

Image analyses were conducted in QuPath using its proprietary image analysis tools (Figure 1).^29^ Manual TMA detection was performed using the ‘Ellipse Annotation’ in conjunction with the ‘Brush’ tool to correct for staining artefacts and tissue damage. The perimeter of cores was excluded in these regions of interest (ROI) to avoid the ‘edge’ effects (non-specific tissue-boundary staining) that were most evident in the t-tau—Opal 570 channel. Quantifications of pathologies and cell types were subsequently performed as an average of values across the two cores from the same standard block (brain region) from each individual.

### Pathology classification

Areal fractions of Aβ and t-tau pathology were quantified with QuPath’s ‘Pixel Classification’ tool using a maximal resolution of 0.5 um/pixel (Figure 1A and B). Thresholds for both pathologies were specific to each standard block from which the cores were obtained. NFTs were manually counted using QuPath’s ‘Multi-Point’ tool.

### Cell detection and classification

Cell quantifications were automated using DAPI signals with QuPath’s ‘Cell Detection’ function. Initially, manual counts were performed with a 22 500 µm^2^ ROI to determine pixel intensity thresholding for cores from each region. Then, iterative automated quantification to match manual counts allowed the thresholding value to be extrapolated to the ROI of these cores.^30^ Sigma values for Gaussian filter and minimum nuclei area were additionally increased to 2 and 10 µm^2^, respectively, to minimize fragmentation of euchromatic nuclei, while all other parameters were defaulted. Classification of cell types was performed with QuPath’s ‘Single Measurement Classifier’ tool. Given the highly ramified morphology of glia, positive cells were classified only if their fluorescence signal colocalized with a DAPI-segmented cell body (Figure 1C-E). This method minimized false positives such as where fragmented processes overlap the nuclei of other cell types. Thresholding of these fluorescence signals was specific to the standard block from which the cores were derived. For IHC, automated NeuN^+^ cell detection was achieved with DAB optical density using a similar manual and automated count matching technique to determine thresholds. In both mIF and chromogenic analyses, automated counts and classifications were manually checked for accuracy.

### Statistical analysis

Notwithstanding the small sample size in this pilot study, potential data trends between case and control tissues were explored using GraphPad Prism (version 10.3.1). Global comparison between case and control tissues was done using unpaired parametric Welch’s one-tailed t-test (*α* = 0.95). For comparison between regions, multiple Welch unpaired t-tests with the Holm-Šídák correction for multiple comparisons were carried out.

## RESULTS

This study investigated the use of TMAs and mIF as a rapid research method for assessing global AD pathologies together with the density of neurons and glial cells. Each TMA contained 35× 2 mm cores consisting of two overlapping cores from the GM, an adjacent and deeper WM core for two neocortical regions: prefrontal cortex (PFC) and parietal cortex (PC) and primary motor cortex (PMC); two GM and an adjacent WM cores, for the occipital cortex (OC), superior temporal cortex (STG), inferior temporal cortex (ITG) and anterior cingulate cortex (ACC), entorhinal cortex (EC), the cerebellar vermis (CV) and cortex (C) and one core from anterior, middle and posterior pons. An iterative mIF process with HIER antibody stripping between was effective in identifying the six pathological and cellular targets across eight TMAs from AD cases (*n* = 4) and controls (*n* = 4). Core loss via tissue delamination was observed after the 5th HIER cycle, although minor delaminations (of cores from the slide) were observed as early as the 3rd cycle of HIER. In general, immunolabelling and DAPI nuclear staining were attenuated in archival tissues (>10 years fixation time before paraffin embedding), affecting cell detection and quantifications.

### Aβ burden across the brain

**Figure 2.**
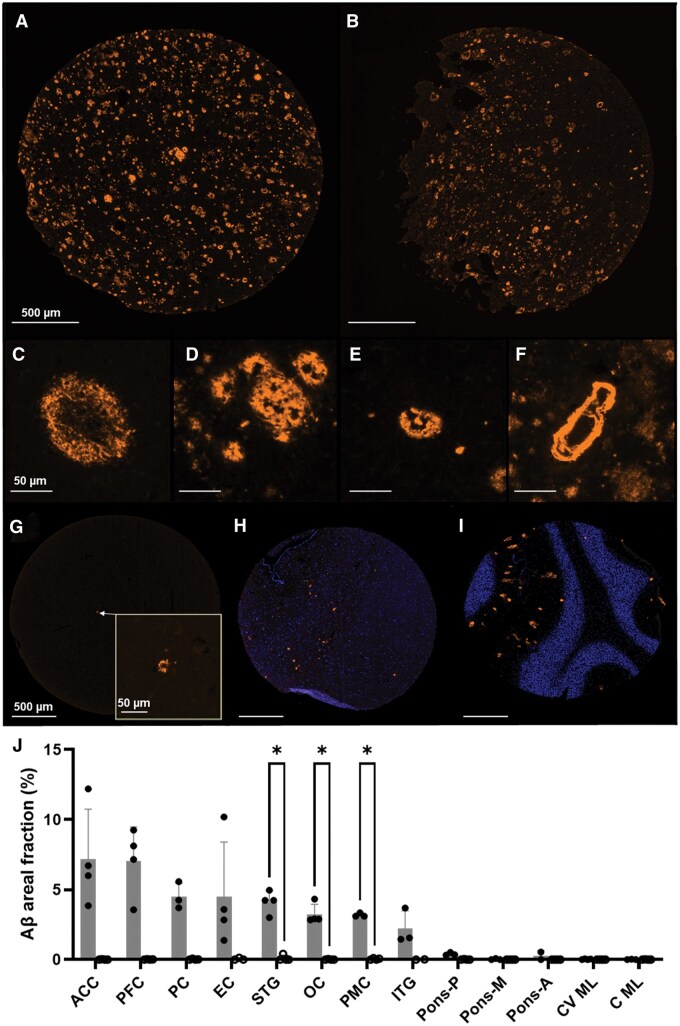
Aβ burdens in AD brain TMA cores. Aβ antigenicity is unaffected by prolonged tissue fixation time as seen between (A) standard 24-hour of an ACC and (B) 17-year fixed STG core. Aβ 1-16 paired with Opal 620 labelled (C) diffuse, (D) compact, (E) cored plaques, and (F) cerebral amyloid angiopathy surrounded by diffuse plaques. Amyloid plaque(s) labelled in a PFC cortical core belonging to a (G) control case, (H) posterior pontine, and (I) the molecular layer of the cerebellum vermis. (J) Ordered (ranked) Aβ areal fractions (±SD) across 11 different cortical regions between AD (grey bars) and control (white bars) tissues. PFC: prefrontal cortex; PC: parietal cortex; OC: occipital cortex; EC: entorhinal cortex; STG: superior temporal gyrus; ITG: inferior temporal gyrus; ACC: anterior cingulate cortex; PMC: primary motor cortex; pons-A/M/P: anterior/middle/posterior; C(V): cerebellum (vermis); ML: molecular layer.

Aβ immunostaining labelled plaque burdens regardless of tissue fixation time (Figure 2A and B). The various plaque subtypes, including diffuse, compact, cored plaques, and cerebral amyloid angiopathy (CAA) were quantified across all cores (Figure 2C-F). CAA was limited to a single AD case (AD2). Aβ immunostaining was negligible in controls including C1 (A1B1C0), though notably, a single plaque was observed in the EC of C4 whose diagnostic score was A0B0C0 (Figure 2G). Across all AD cases, plaques were observed in both anterior (0.39%) and posterior (0.27%) pons cores (Figure 2H), and additionally in the molecular layer of the cerebellum vermis of AD2 (Figure 2I).

Mean Aβ areal fractions across all regions differed between AD cases (3.1%) and controls (0.02%, *P* < .0001) and between the neocortex (4.6%) and subcortical structures (0.13%, *P* < .0001) within the AD cohort (Figure 2J). Regional maximal Aβ areal fractions were observed in the ACC at ∼7.2% and declined throughout the neocortex and the EC to a minimum of 2.2% in the ITG. The ACC, PFC, and EC showed the greatest variability between the four cases. Specifically, an 8.8% difference was observed between maximal and minimal areal fractions seen in EC. Differences for the remaining regions were minimal (< ±1.5% SD) with STG and PMC exhibiting the lowest variability between cases (< ±0.5% SD).

### Tau burdens across the brain

**Figure 3.**
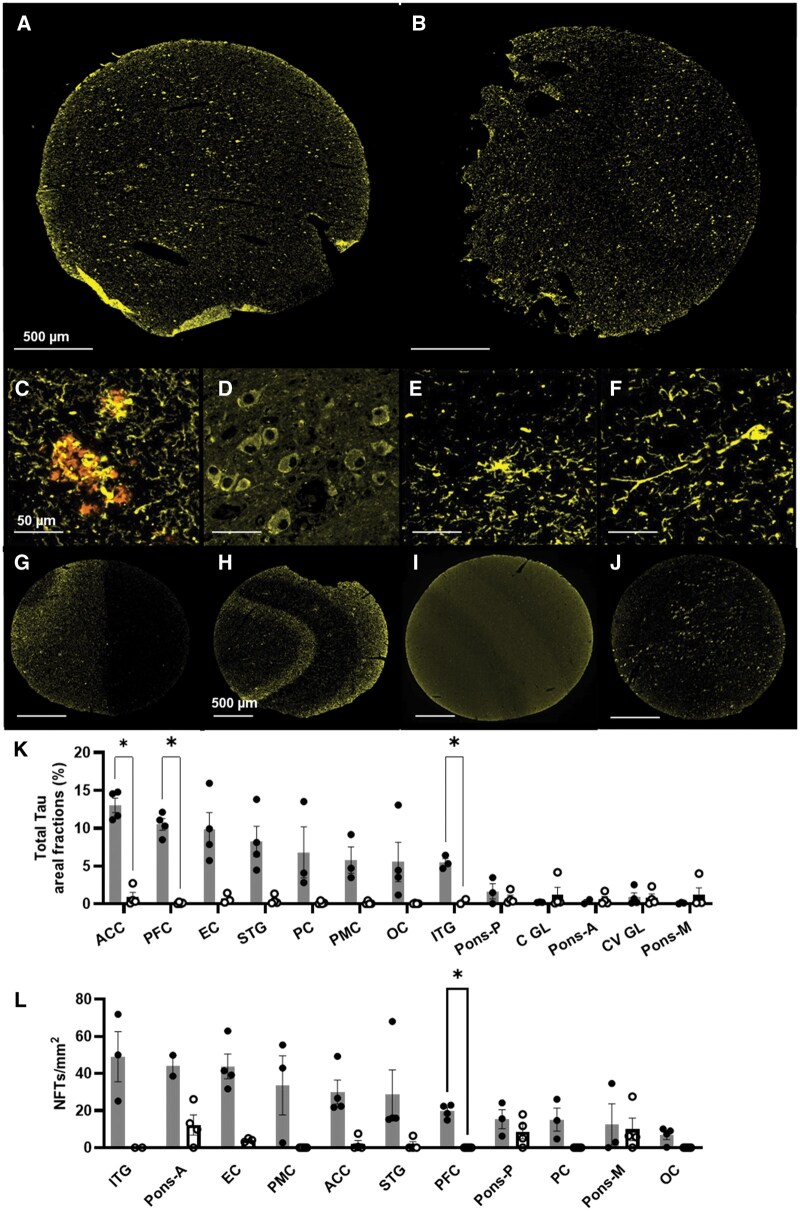
Pathogenic tau in AD brain TMA cores. T-tau immunolabelling is unaffected by tissue fixation time with both (A) standard 24-hr and (B) 17-year fixed tissues stained on the same TMA showing comparable staining. In AD tissues, t-tau labelled (C) neuritic plaques co-labelled with Aβ 1-16, (D) pre-tangles within the pons, (E) neuropil threads and (F) NFTs. (G) GM-WM boundary and (H) line of Gennari in the OC are delineated by tau features. (I) Control PFC cortex with enhanced pixel values thresholding showing minimal t-tau immunoreactivity. (J) NFTs and pre-tangles are numerous in the anterior pons. (K) Ordered NFT density and (L) t-tau areal fractions across regions between cases (grey bars) and controls (white bars). PFC: prefrontal cortex; PC: parietal cortex; OC: occipital cortex; EC: entorhinal cortex; STG: superior temporal gyrus; ITG: inferior temporal gyrus; ACC: anterior cingulate cortex; PMC: primary motor cortex; pons-A/M/P: anterior/middle/posterior; C(V): cerebellum (vermis); ML: molecular layer; GL: granular layer.

T-tau signals were observed across the 11 regions sampled in AD and control cases, and similarly showed negligible attenuation with increasing fixation time (Figure 3A and B). In AD, multiple types of tau pathologies were labelled in the cortex, ranging from NP, pre-tangles, NT, and NFTs (Figure 3C-F). NTs were the most abundant form of neurofibrillary pathology and clearly demarcated the boundary between GM and WM, and the myelinated axons within the line of Gennari in the OC (Figure 3G and H; also see Figure S4A for AT8-tau staining of these features). These features were absent in the grey matter of controls (Figure 3I), although tau pathologies were present within their cerebral WM and cerebellar granular layer. In addition, tau-immunoreactive neurons (‘pre-tangles’) were observed throughout the cortex but were most prevalent within the neurons of pontine nuclei in both controls and cases (Figure 3J).

T-tau areal fractions differed between case (5.8%) and control cores (0.57%, *P* < .0001) (Figure 3K). The maximal average tau areal fraction was observed in the ACC at 13.03% and incrementally decreased in the neocortex to the minimum of 5.5% of the ITG. Overall, regions such as the OC showed ∼12% difference between the four AD cases, while areas such as the ACC, PFC, and ITG showed closer concordance. NFT density also differed between cases and controls, but this was not correlated with t-tau areal fractions (Figure 3L). Indeed, the ITG exhibited the highest mean count, 49 NFTs/mm^2^, and contrasted with the sparsest NFTs in the OC at 5.5 NFTs/mm^2^, despite the two showing comparable tau areal fractions of ∼5.5%. This dearth of NFTs in the OC was not a consequence of our antibody choice, as substantive neurofibrillary pathology without NFTs was also demonstrated with AT8-tau antibody, showing NPs, DN, and NT (File S4A-C).

In deeper structures, pons and cerebellar tau areal fractions were measured at <1% except for the posterior pons core (1.7%). NFT density was highest in the anterior pons, measuredly like the ITG. In contrast, ACC, EC, STG, PMC, ITG, and posterior pontine cores had lower NFT densities, but all showed high variability with a > 20 NFTs/mm^2^ spread across the four cases. There was a closer concordance between Aβ and t-tau areal fractions with pathologies highest in the ACC and PFC, and lowest in the ITG (Figures 2J and 3L)—the site of the highest NFT density.

### Cell quantification

The immunoreactivity of markers for microglia (ie, Iba1), astrocytes (ie, GFAP), and neurons (ie, NeuN) was affected by tissue fixation time. The use of archival tissues (22% of all cores) attenuated their detection and subsequent quantification. Similarly, DAPI nuclei labelling was significantly impeded and non-existent in archival tissue, leading to cores with glial and neuronal labelling, but without any nuclei for segmentation.

### Iba1^+^ cells

**Figure 4.**
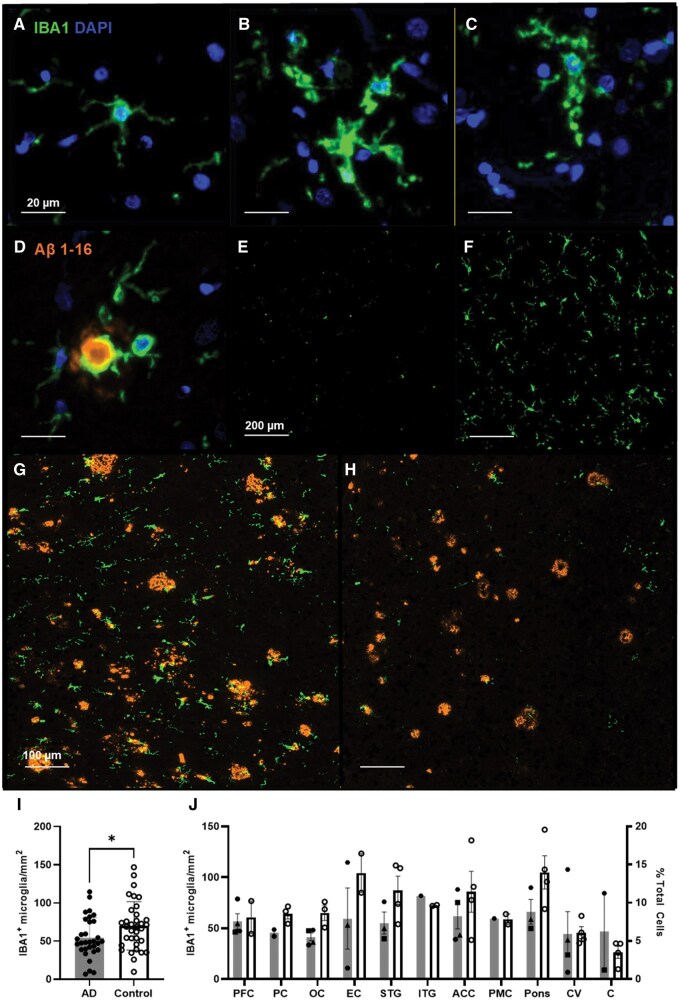
Iba1^+^ microglia in the AD and non-demented human brain. A variety of Iba1^+^ microglia phenotypes are detected across the breadth brain regions sampled by the TMAs, including (A) ramified microglia from a control tissue, (B) activated microglia exhibiting microgliosis, (C) dysfunctional phenotype with disrupted processes and spheroidal swellings, and (D) activated microglia with process surrounding a small amyloid plaque. Iba1 immunoreactivity is affected by fixation time as shown between (E) archival and (F) standard fixed STG cores. Further variability in Iba1 expression observed seen from two (G) and (H) different cores obtained from the same OC block. (I) Overall Iba1^+^ cell densities across regions between cases (grey bars) and controls (white bars). (J) Regional comparison of Iba^+^ cell counts, and their fraction of total cells (±SD) detected. PFC: prefrontal cortex; PC: parietal cortex; OC: occipital cortex; EC: entorhinal cortex; STG: superior temporal gyrus; ITG: inferior temporal gyrus; ACC: anterior cingulate cortex; PMC: primary motor cortex; pons-A/M/P: anterior/middle/posterior; C(V): cerebellum (vermis); ML: molecular layer; GL: granular layer.

Different Iba1^+^ cell (microglial) morphologies were detected across both healthy and diseased tissues, including those traditionally described as ramified, activated, dystrophic with fragmented processes, and cells in direct interactions with plaques (Figure 4A-D). Dystrophic microglia exhibited both attenuated immunoreactivity to Iba1 in addition to a sparser distribution (Figure 4E) compared to healthy cells (Figure 4F). Notably, this study observed cores obtained from the same donor tissue block showing both the typical activated microglia phenotype and distribution in response to plaques (Figure 4G) as well as dystrophic phenotypes (Figure 4H).

Mean Iba1^+^ cell density across the 11 regions were higher in controls (∼70 cells/mm^2^) compared to cases (∼54 cells/mm^2^, *P* = .02) (Figure 4I). Only modest inter-cohort differences were seen in the EC, STG, ACC, pons, and cerebellum (Figure 4J). Average density of Iba1^+^ cells was highest in the EC and pons for both cases and controls at ∼83 cells/mm^2^, followed by the remainder of the neocortex at ∼64 cells/mm^2^, and the lowest counts were observed for the cerebellar cores of ∼41 cells/mm^2^. Overall, the microglial fraction ranged from 6% to 12% of the total number of cells (all DAPI-nuclei) detected.

### GFAP^+^ cells

**Figure 5.**
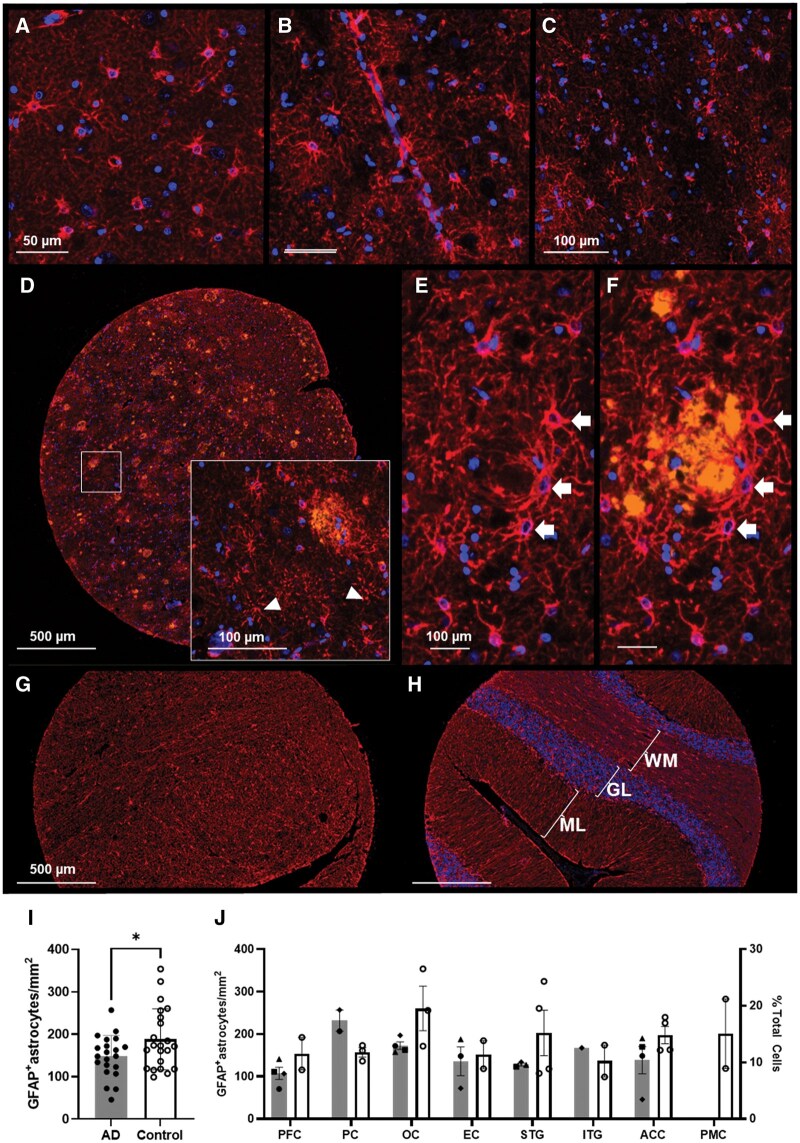
Astrocytes immunolabelling with GFAP and Opal 690. (A) GFAP immunolabels astrocyte soma, processes, (B) perivascular end feet. (C) Regions of low GFAP signals containing no astrocyte soma and few processes are observed in both AD and control tissues. (D) Aβ 1-16^+^ and GFAP^+^ signals in an AD core, showing astrocyte soma and processes labelled with GFAP but no nuclei. (E) and (F) A cluster of GFAP^+^ astrocytes in interaction with a plaque with processes both enveloping and penetrating the pathology. GFAP is highly expressed throughout the (G) pons and (H) cerebellum, however, distinct astrocyte soma are difficult to dissect. (I) Overall GFAP^+^ cell densities across regions between cases (grey bars) and controls (white bars). (J) Regional GFAP^+^ cell densities and their respective fraction of the total detected cells (±SD). AD1-4 are represented by filled triangle, circle, square, and diamond. PFC: prefrontal cortex; PC: parietal cortex; OC: occipital cortex; EC: entorhinal cortex; STG: superior temporal gyrus; ITG: inferior temporal gyrus; ACC: anterior cingulate cortex; PMC: primary motor cortex; ML: molecular layer; GL: granular layer; WM: white matter.

Astrocytes showed strong GFAP immunoreactivity in their soma and perivascular end feet that then tapered off in the distal processes (Figure 5A). Across all tissues, automatic quantification of astrocytes was prone to false positives where the GFAP^+^ processes overlapped with other nuclei, such as those presumed to be vascular pericytes (Figure 5B). Counts were further affected by regions of attenuated GFAP^+^ signals and areas where the cell soma and processes were labelled but not their nuclei (Figure 5C). Like microglia, regions of attenuated GFAP immunoreactivity were observed where both cell soma and processes were undetectable (Figure 5D). In the AD cohort, astrogliosis was observed across all cortices containing Aβ plaques, characterized by hypertrophic soma and proximal processes. These cells are observed in clusters enveloping and occasionally penetrating plaques with their processes (Figure 5E and F). GFAP^+^ astrocytes were present throughout pontine and cerebellar cores; however, individual astrocyte cell bodies were difficult to discern with automation (Figure 5G and H).

Global GFAP^+^ counts were higher in controls (∼188 cells/mm^2^) compared to cases (∼147 cells/mm^2^, *P* = .019) and accounted for 10%-20% of total DAPI-nuclei detected across all donors (Figure 5I and J). However, regional densities were not significantly different between cases and controls, although the means for PFC, OC, STG, and ACC were marginally higher in controls, but the reverse was observed for PC. Maximum astrocyte density was seen in the OC, STG, and PMC (notwithstanding the loss of cores in the AD cohort) at ∼200 cells/mm^2^. For the remainder of the neocortex and the EC, astrocyte densities ranged from 140 to 160 cells/mm^2^ with the minimum of 130 cells/mm^2^ in the PFC.

### NeuN^+^ cells

**Figure 6.**
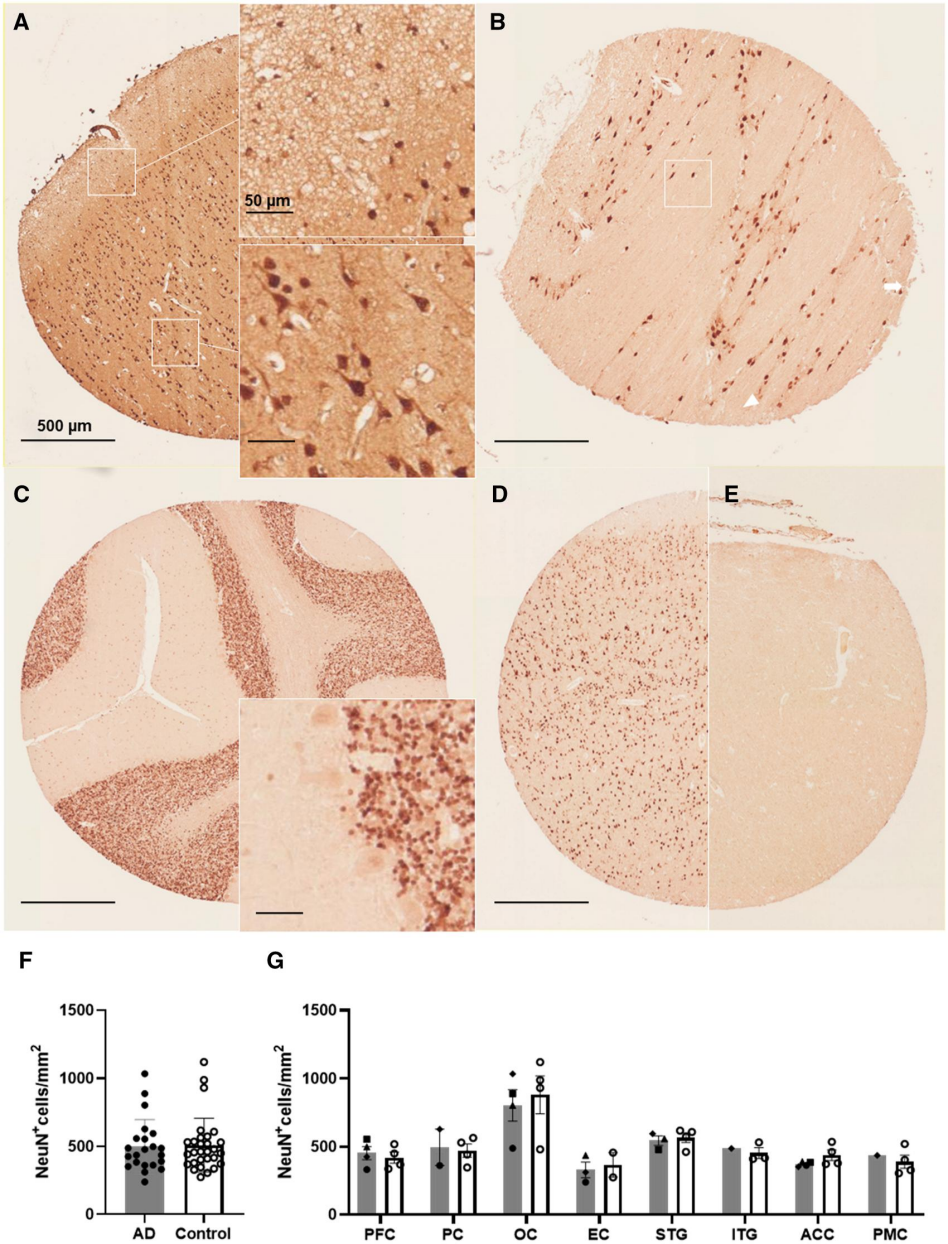
Detection of neurons using anti-NeuN with chromogenic DAB in TMAs. NeuN immunolabelling of different neurons throughout the cortex including (A) superficial neurons in layers I-II, pyramidal and interneurons, (B) neurons between the corticospinal tracts in the Pons, (C) granule neurons of the cerebellum. NeuN immunoreactivity is affected by tissue fixation time as distinct neuronal bodies are observed for (D) standard fixed and entirely non-existent for (E) archival STG cores. (F) Overall NeuN^+^ cell densities across regions between cases (grey bars) and controls (white bars). (G) NeuN^+^ cell densities across different regions of the AD and non-demented human brains. PFC: prefrontal cortex; PC: parietal cortex; OC: occipital cortex; EC: entorhinal cortex; STG: superior temporal gyrus; ITG: inferior temporal gyrus; ACC: anterior cingulate cortex; PMC: primary motor cortex.

Chromogenic staining of NeuN labelled all neuronal cell types throughout the cortical lamina, neurons within pontine nuclei, the cerebellar granule neurons, but not the Purkinje cells (Figure 6A-C). Anti-NeuN immunoreactivities were affected by tissue fixation time, with all archival cores (nearly one third of all GM cores) showing low or negligible neuronal labelling (Figure 6D-E). This impeded comparisons between AD and control tissues. Nevertheless, our chromogenic data revealed that NeuN^+^ cell counts varied across regions like glial counts but did not globally differ between cases (∼502 cells/mm^2^) and controls (∼508 cells/mm^2^, *P* = .46) (Figure 6F). Maximum neuronal density was seen in the OC (842 NeuN^+^ cells/mm^2^), followed by relatively similar densities for the PFC, PC, STG, ITG, ACC, and PMC (∼460 NeuN^+^ cells/mm^2^) (Figure 6E). The EC had a lower density (347 cells/mm^2^) while the sparsest neurons were observed in pontine regions with an average of 115 cells/mm^2^.

## DISCUSSION

TMA is a high-throughput platform typically used for assessing biomarkers across a variety of tissue types,^31^ or assaying their expression across large cohorts.^22^^,^^23^ The one previous study that employed the technique across multiple brain regions relied on IHC staining of adjacent sections and labor-intensive manual assessment.^25^ In this study, we showed that the integration of a 2 mm core TMA sampling schema, mIF, and a semi-automated quantification workflow can simultaneously assess the severity of AD pathologies and quantify different cellular lineages from multiple regions across a single human brain. This allows the regional distribution of AD hallmark pathologies to be empirically determined, and differentially affected regions subsequently selected, for more focused research studies to explore the cellular and molecular impact of varying ADNC loads.

TMA quantified Aβ areal burdens are comparable with data obtained from the classical whole tissue section (WTS) approach.^32^ Aβ areal fractions of the OC, ITG, and PFC were congruent to a previous WTS study using different BTRC cases, with burdens for the OC and ITG comparably both below 5%.^16^ PFC values were higher in TMAs than data from WTS, though both studies showed greater inter-case variability in this region compared to others. TMA and WTS data were also comparable with IHC-autoradiography, which showed a 4%-15% variability across cases in the ACC.^33^ Similarly, plaque burdens in the frontal (BA9), temporal (BA22), and parietal (BA39) cortices of both diabetic and non-diabetic AD cases (Braak V-VI) ranged from 2.7% to 5.6% and were equivalent to our data.^34^

Overall, our TMAs measured the variability of Aβ burden across different brain regions to be consistent with the established spread of plaques.^32^ We observed a decrease from the ACC and PFC to the ITG, which aligns with the known spread of Aβ within the neocortex over the disease course. Recent imaging studies using Pittsburgh Compound B have demonstrated these similar regional trends, with higher loads in the frontal and parietal regions to the lowest in the temporal gyri.^35–37^ Given that plaques are relatively homogeneously distributed across the cerebral gyri,^38^ our TMA data likely recapitulate the varied plaque burdens across these regions and also provide a reference to inform region selection for subsequent investigations. However, additional investigation is required to determine if the presence of the cerebellar plaques in the singular AD case is representative of the rarity of ADNC there, or rather an artefact of an insufficient coring schema to capture its more heterogenous distribution.

Neurofibrillary pathology in AD is typically assessed by quantifying just NFTs. This is due to the presumption that they precede neuronal loss and that their discrete nature rather than expressions are amenable to quantification.^39^ Nonetheless, a previous study has shown that NTs are at least as frequent as NFTs.^40^ Hence, we opted to measure tau by its areal fraction to quantify all tau-immunopositive features such as pre-tangles, NPs, NTs, and NFTs.^41^ Using a t-tau antibody,^42^ we show that the abnormal tau areal fraction differed between cases and controls. AD cases displayed an abundance of tau-immunopositive structures consistent with Braak stage VI,^43^ while controls exhibited the regional pattern that is consistent with primary age-related tauopathy.^44^^,^^45^ Interestingly, the expected pattern of tau accumulation outlined by Braak’s staging scheme was only reproduced by NFT densities but not t-tau areal fractions for the AD cases. Specifically, we saw greater areal burdens in two neocortical regions, the ACC and PFC, over the EC, contradicting the established stereotypical spread.^43^ The remainder 5 cortical regions, the STG, PC, PMC, OC, and ITG, all exhibited lower burdens than the EC, and are consistent with stages V and VI. The difference observed was unlikely due to our use of a t-tau antibody. We and colleagues have demonstrated the high overlap with a p-tau (AT8) antibody.^41^ Indeed, NFT densities here, commonly detected with p-tau, align with Braak staging across all regions except for the ITG. The t-tau antibody has also been used to show pathologies arising from other avenues of neuronal damage, including traumatic brain injuries, Creutzfeldt-Jakob disease, and strokes.^46–48^

A major knowledge gap in AD pathogenesis, and one seemingly at odds with the linear chain of events suggested by the amyloid cascade hypothesis, is why NFTs and Aβ plaques have quite disparate distributions.^2^^,^^32^ Prior studies have also shown neuronal loss to vastly exceed NFT densities of both hippocampal CA1 and superior temporal sulcus, suggesting that alternative mechanisms of neuronal degeneration are indeed at play.^49^^,^^50^ The congruency between t-tau and Aβ areal burdens here suggests a much wider impact of Aβ on neuronal damage through neurofibrillary pathology and not restricted to NFTs.

When comparing with prior WTS work using different BTRC tissues, we found WTS areal fractions were 2-5 times lower across regions analogous to our PFC, ITG, and OC.^16^ However, congruency between TMA and WTS was achieved in the NFT densities of the OC and PFC, at ∼10 and 20 NFTs per mm^2^, respectively. Whilst our ITG values are higher than their WTS equivalent, both studies showed high variability that exceeded 40 NFTs per mm^2^. From our preliminary data, TMA may be seen as a viable platform to assay AD tauopathy when only NFT densities are examined. However, more studies are required to support the ability of the platform to recapitulate the full breadth of neurofibrillary pathologies.

Concomitant to pathology in AD are the changes to the different cell types in the brain. We showed here that morphological changes to astrocytes and microglia in AD tissues are consistent with their reported activated phenotypes in response to disease.^51^ Our data indicate that microglia account for 6%-12% of total cells detected across regions, in line with the ∼9% values previously established in healthy tissue using a combination of markers such as Iba1, MHC-II, and CD68.^52^ In absolute values, mean Iba1^+^ counts were 40%-60% lower than values from WTS of the OC, PFC, ITG, ACC, and ITG obtained using abridged stereological quantification techniques.^16^^,^^53^ Astrocyte counts in the PFC were 28% lower than values obtained using 5 µm WTS sections with GFAP,^54^ but surprisingly comparable to those obtained from a stereological approach when the dissector height was normalized for both the PFC (∼148.7 cells/mm^2^) and OC (∼176 cells/mm^2^).^55^ Here, the reduced size and section thickness of TMAs limit the number of cells captured, and therefore affected by the highly mobile and heterogeneously distributed microglia and both microglia and astrocytes with their large three-dimensional non-overlapping domains.^9^

In contrast to both glia, neuronal counts were 40%-50% lower in the OC, PFC, and ITG from WTS obtained previously with Cresyl violet staining.^16^ Higher counts in TMAs are likely attributable to the areal constraints and differences in staining techniques. Previous studies using both human and rat tissues showed that NeuN detected 16%-32% more pyramidal and non-pyramidal cells over the histological Cresyl violet staining.^56^^,^^57^ Importantly, however, the regional pattern of neuronal densities across these neocortical regions was similar, with the highest seen in the OC followed by both PFC and ITG. This indicates that the TMA sampling schema can adequately portray the relative abundance of neurons across regions.

The overall trend of higher glial densities in control tissues suggests they are negatively impacted throughout the AD brain. However, the limited sample size combined with loss of data stemming from delaminated cores in this pilot study meant insufficient power to identify significant regional differences. We have previously shown that microglia densities were regionally affected between cases and controls.^16^ Here, comparable densities between AD cases and controls were found for both PFC and OC, though microglia in the ITG of AD cases were 37% lower. In a murine model, the interaction between microglia and phospho-tau drove their degeneration and death, resulting in morphologies like the low-immunoreactive Iba1^+^ cells observed here.^58^ Similarly, the decrease in GFAP immunoreactivity observed across AD tissues may indicate the indirect effects of Aβ-mediated immune response. Indeed, TNF-*α* treated astrocyte cultures exhibited elevated serum GFAP but at a reduced cellular expression.^59^ However, the use of GFAP as the prototypical pan-astrocytic marker may have also created an unintended bias.^60^ A recent comparison of several astrocyte markers in BTRC tissues, including GFAP, showed 30%-60% cases with GFAP-immunonegative cells in layer III-VI of the superior frontal cortex.^61^ With improving iterative mIF techniques, the addition of complementary markers such as ALDH1L1, GLT1, Cx43, S100β, and AQP4 in higher multiplex investigations will determine whether the low GFAP^+^ expression results from astrocyte loss, disease-mediated GFAP downregulation, or a GFAP-immunonegative ‘resting’ phenotype.^61^ Similarly, disease-associated markers for microglia such as TREM2, CD68, CD33, CD14, and OPN (SPP1) can shed light on the dynamic responses of microglia to AD hallmarks when Iba1 expression is attenuated or lost.^52^^,^^62^ In this scenario, TMA offers a powerful platform to allow simultaneous and global assessment of both the phenotypic proportion of these different cell lineages, in addition to their morphological response, similar to the immune profiling approaches of oncological studies.^63^

To our knowledge, no IF studies have previously attempted to simultaneously quantify AD hallmarks and the abundance of different cell types across 11 regions of the brain using TMAs. Our results indicate that a 2× 2 mm core sampling schema can globally assess the variable severity of Aβ plaques and NFTs in the human brain and provide metrics that corroborate with both pathogenic spreading patterns from Braak and Thal.^2^^,^^3^ We further show that although TMA cell counts obtained with an automated non-stereological approach are incomparable with traditional stereological studies, the TMAs offer a useful tool to examine the relative abundance of cell subtypes across multiple regions.

Overall, our data provide a reference for future research projects, enabling the selection of regions based on their ADNC burdens. Subsequent experiments could target incrementally affected regions and utilize interregional comparisons in a larger cohort to model the natural history of the disease. When combined with molecular techniques, the resulting proteins, transcripts, and pathways can be correlated to increasing Aβ or tau burden, or be found to be independent of either. Specifically, we predict that the least affected areas of the postmortem AD brain may harbor early and reversible changes that could form the basis of new neuroprotective therapeutic targets. Moving forward, TMAs can be further refined by including more cell subtype-specific markers given their continual identification by single cell technologies. TMA and mIF can also be incorporated into spatial transcriptomic workflows to maximize sampling across different regions and cases in the current size-constrainted platforms. Together, these two technologies will permit the quantification of different cell subtypes, their interactions with other cells (subtypes and lineages), and the pathological hallmarks to define a new future for AD neuropathology.

## Supplementary Material

nlaf064_Supplementary_Data

## Data Availability

All data are contained within the manuscript, figures, and supplementary files.
